# Regulatable Orthotropic 3D Hybrid Continuous Carbon Networks for Efficient Bi-Directional Thermal Conduction

**DOI:** 10.1007/s40820-024-01426-0

**Published:** 2024-05-17

**Authors:** Huitao Yu, Lianqiang Peng, Can Chen, Mengmeng Qin, Wei Feng

**Affiliations:** School of Materials Science and Engineering, Tianjin Key Laboratory of Composite and Functional Materials, Tianjin University, Tianjin, 300350 People’s Republic of China

**Keywords:** Orthotropic continuous structures, Hybrid carbon networks, Carbon/polymer composites, Thermal interface materials

## Abstract

Vertically oriented carbon structures constructed from low-dimensional carbon materials are ideal frameworks for high-performance thermal interface materials (TIMs). However, improving the interfacial heat-transfer efficiency of vertically oriented carbon structures is a challenging task. Herein, an orthotropic three-dimensional (3D) hybrid carbon network (VSCG) is fabricated by depositing vertically aligned carbon nanotubes (VACNTs) on the surface of a horizontally oriented graphene film (HOGF). The interfacial interaction between the VACNTs and HOGF is then optimized through an annealing strategy. After regulating the orientation structure of the VACNTs and filling the VSCG with polydimethylsiloxane (PDMS), VSCG/PDMS composites with excellent 3D thermal conductive properties are obtained. The highest in-plane and through-plane thermal conductivities of the composites are 113.61 and 24.37 W m^−1^ K^−1^, respectively. The high contact area of HOGF and good compressibility of VACNTs imbue the VSCG/PDMS composite with low thermal resistance. In addition, the interfacial heat-transfer efficiency of VSCG/PDMS composite in the TIM performance was improved by 71.3% compared to that of a state-of-the-art thermal pad. This new structural design can potentially realize high-performance TIMs that meet the need for high thermal conductivity and low contact thermal resistance in interfacial heat-transfer processes.

**Supplementary Information:**

The online version contains supplementary material available at 10.1007/s40820-024-01426-0.

## Highlights

- A composite thermal interface material with three-dimensional hybrid carbon network-reinforced polydimethylsiloxane was proposed.
- The cooperative regulation of thermal conductivity and mechanical properties was achieved by controlling the assembly process of micro-nano scale unit carbon materials.
- The orthotropic continuous carbon structure endowed the composites with in-plane and out-of-plane thermal conductivities of up to 113.61 and 24.37 W m^−1^ K^−1^, respectively.
- The excellent compressibility and adhesion properties cooperatively improved the effective thermal conductivity by more than an order of magnitude.

**Supplementary Information:**

The online version contains supplementary material available at 10.1007/s40820-024-01426-0.

## Graphical abstract

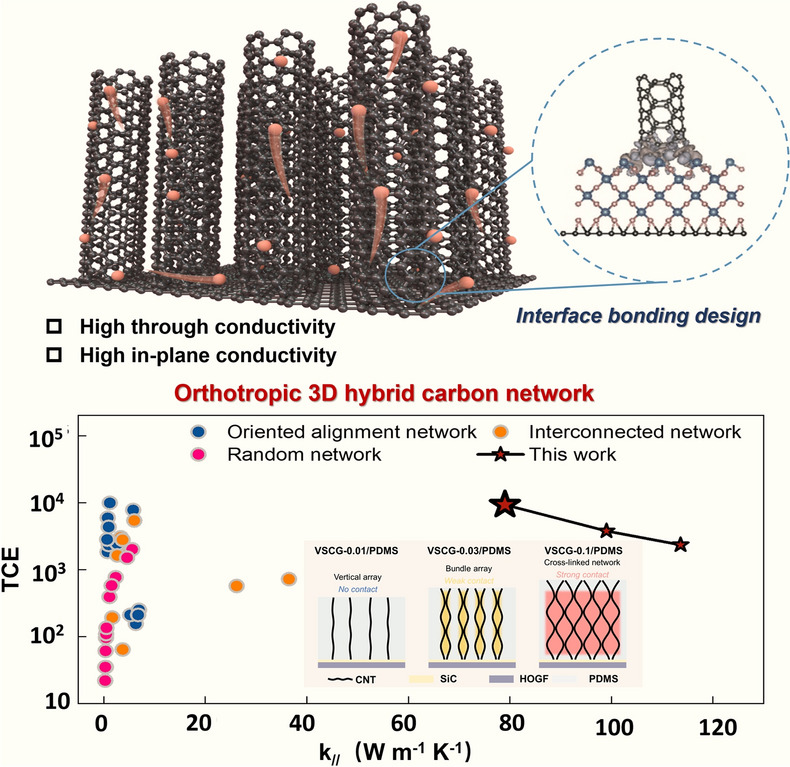

## Introduction

Guided by Moore’s law, devices in the consumer electronics industry and the military field are continually evolving toward miniaturization, modularity, and integration [1]. However, the performance of high-power density devices is usually limited by the interface between the components, which controls the temperature of the device. The heat-transfer efficiency across the interface in a thermal-management system is usually poor [2], leading to hot spots that decrease the operational stability, efficiency, and lifetime of the device and sometimes cause thermal failure. To resolve these problems, advanced high-performance thermal interface materials (TIMs) with high thermal conductivity and good mechanical property are urgently demanded [3–5]. Polymer materials are rheologically suitable for TIMs because they are outstandingly flexible, compression resilient, and adhesive, facilitating strong phonon–electron coupling at the solid–solid interface [6–8]. Unfortunately, improving the mechanical adaptability of polymeric materials inevitably decreases their intrinsic thermal conductivity [9]. Alternatively, polymers can be compounded with thermally conductive fillers using conventional solution or melt-blending methods, but the thermal enhancement of this approach is limited by agglomeration effects; moreover, high filling levels can damage the rheology of the composite [10, 11]. Therefore, transferring a high interfacial heat flux is severely challenged by the tradeoff between thermal conductivity and mechanical property of implanted TIMs.

The tradeoff problem can be feasibly solved by constructing flexible networks of highly thermally conductive fillers and compounding them with rheologically favorable polymers [7, 12–15]. Networks derived from low-dimensional carbon materials (graphene and carbon nanotubes (CNTs)) with superior thermal conductivity and deformability are ideal frameworks for polymer-based TIMs [16]. However, the assembly method can degrade the comprehensive thermal conductivity of such composites because of the low-dimensional carbon materials showing anisotropic thermally conductive properties. Although isotropic 3D carbon networks of fillers endow the polymer matrix with homogeneous heat-transfer pathways, their low-density porosity increases the contact thermal resistance at the junctions formed by physical overlap of the low-dimensional carbon materials. High contact resistance prevents the effective transfer of phonons and stresses within the composites [17–19]. Low-dimensional carbon materials can also be aligned into oriented 3D carbon networks that enhance the mean free range of phonon vibrations, but the weak interactions perpendicular to the alignment direction lead to inhomogeneous heat distribution inside the composites and limit the through-plane heat transfer at rough interfaces [8, 12, 20]. Consequently, thermally conductive carbon networks are dimensionally dependent and the cross-order-of-magnitude behavior of the thermal conductivity depends on the porosity and orientation of the micro-nano carbon structures. By optimizing the micro-nano structure (such as densification degree, orientation, and surface interface) of low-dimensional carbon materials, we could construct efficient multi-level phonon transfer pathways that synergistically improve the 3D thermal conductivity of polymer-based TIMs.

The present study introduces an orthotropic 3D hybrid carbon network (VSCG) with a regulated oriented structure and a fine interface design. The orthotropic VSCG structure was obtained by silane-functionalized modification on the surface of a horizontally oriented graphene film (HOGF), deposition of CNT on its surface by floating catalytic chemical vapor deposition method (FCCVD), and annealing strategy. The VSCG micro-nano structure was dynamically regulated by controlling the CNT growth conditions. Furthermore, a standalone flexible, adhesive, highly thermally conductive TIM (VSCG/PDMS) was developed by homogeneously compounding VSCG (a thermally conductive framework) with a polydimethylsiloxane (PDMS) matrix using a vacuum-assisted impregnation technique. The VSCG formed orthotropic 3D interconnected thermally conductive pathways, which are prerequisite to efficient thermal conduction. Meanwhile, the excellent flexibility of VSCG and PDMS synergistically improved the mechanical properties of VSCG/PDMS. In an actual thermal management performance verification, our proposed VSCG/PDMS composites effectively suppress the “hot spot” problem compared to that of a state-of-the-art commercial TIM, demonstrating their great potential for cooling electronic devices.

## Experimental Section

### Materials

Tetraethyl orthosilicate (TEOS) and xylene were obtained from Aladdin Co., Ltd. Ferrocene was purchased from Alfa Aesar Co. Ltd. Polydimethylsiloxane (PDMS) resin was purchased from Dow Corning Co., Ltd. All the chemicals were used without further purification. Deionized water was prepared in the laboratory.

### Material Preparation

#### ***Fabrication of the SiO***_***2***_***-HOGF Substrate***

The graphene oxide (GO) prepared by the modified Hummers method was configured as a homogeneous GO aqueous dispersion of 1 mg mL^–1^ [21]. The dispersion was assembled into the GO film (GOF) by direct vacuum filtration through an anodic Al_2_O_3_ filter membrane (Anodisc 47; Whatman) with the pore size of 0.45 μm. Subsequently, a layer of TEOS was uniformly spin-coated on the surface of the GOF. After natural drying, it was sandwiched between graphite plates and heated in a tube furnace, allowing TEOS to be pyrolyzed to generate SiO_2_ and adsorbed on the surface of the HOGF derived from GOF reduction, obtaining SiO_2_-modified HOGF (SiO_2_-HOGF). During the pyrolysis, the temperature of the furnace was elevated to 1000 °C at a heating rate of 10 °C min^–1^ and held for 1 h, and the pure H_2_ and Ar were flown at rates of 20 and 200 sccm.

#### Fabrication of the VSCG Hybrid Network

VSCG hybrid networks were synthesized by FCCVD and in situ annealing method. In the FCCVD process, ferrocene powders (catalyst precursor) were dissolved in xylene (carbon source). The precursor solution was injected continuously at a rate of 20 mL h^–1^ by a syringe pump into a tube furnace loaded with a SiO_2_-HOGF substrate. The temperature of the furnace was set as 860 °C, and the carrier gases of pure H_2_ and Ar flow rates were 150 and 1000 sccm. CVD time was fixed at 20 min. After the growth process, the H_2_ was turned off and the Ar flow rate was adjusted to 500 sccm. The furnace was raised to 1700 °C at 10 °C min^–1^ and held for 30 min for annealing, and the VSCG hybrid network was obtained by allowing the furnace to cool naturally to room temperature. In this case, three sets of samples (VSCG-0.01/VSCG-0.03/VSCG-0.1) were prepared with the concentration of ferrocene/xylene solution (0.01/0.03/0.1 g mL^–1^) as the control variable.

#### Fabrication of the VSCG/PDMS Composites

PDMS resin was spin-coated into the interior of the VSCG network. The spinning process was repeated several times to ensure complete penetration of PDMS into the VSCG network. The composite was transferred to a vacuum drying oven for 2 min at 25 °C, then returned to atmospheric pressure for 1 min. This process was repeated many times. After curing at 120 °C for 2 h, three sets of VSCG/PDMS composites (VSCG-0.01/PDMS, VSCG-0.03/PDMS, and VSCG-0.1/PDMS) were obtained.

## Results and Discussion

### Fabrication and Structural Characterization of VSCG/PDMS Composites

**Fig. 1:**
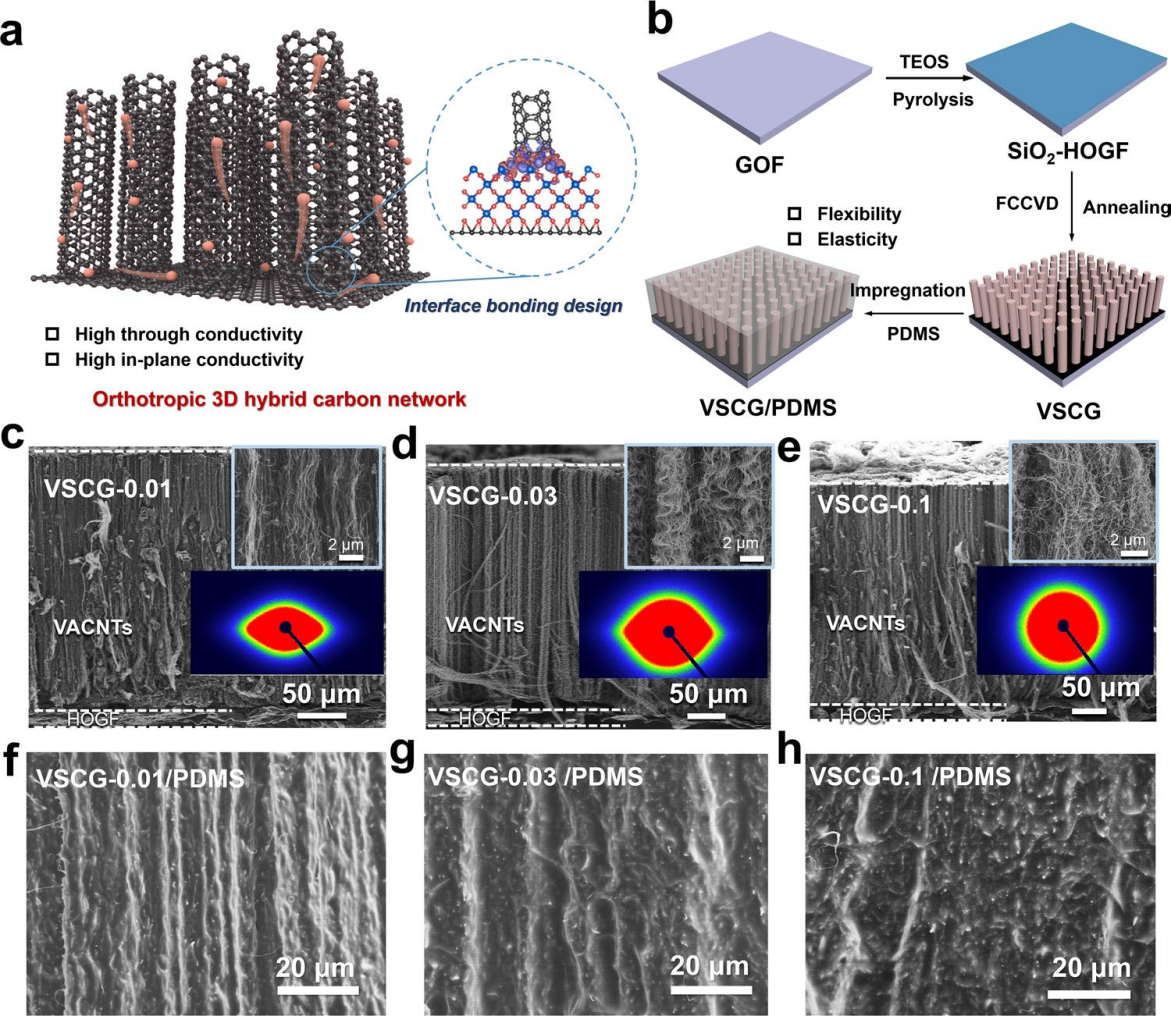
Schematics showing **a** the structural design concept of the VSCG network and **b** the synthetic route of the VSCG/PDMS composite; HOGF: horizontally oriented graphene film; SEM images of VSCG prepared at different catalyst concentrations: **c** 0.01, **d** 0.03, and **e** 0.1 g mL^−1^; 2D SAXS images of the VACNTs within VSCG prepared at catalyst concentrations of 0.01, 0.03, and 0.1 g mL^−1^; cross-sectional morphologies of the composites after PDMS penetration in **f** VSCG-0.01, **g** VSCG-0.03, and **h** VSCG-0.1. VACNTs: vertically aligned carbon nanotubes; TEOS: tetraethyl orthosilicate; VSCG: orthotropic 3D hybrid carbon network; PDMS: polydimethylsiloxane

The HOGF possesses excellent flexibility and high in-plane thermal conductivity, whereas the VACNTs feature good elasticity and high through-plane thermal conductivity [22, 23]. Here, a novel orthotropic 3D hybrid carbon network (VSCG) that combines the superior characteristics of HOGFs and VACNTs was prepared. The HOGF and VACNTs were assembled through the oriented structural regulation and fine interface bonding design (Fig. 1a). The VSCG network was tailored to an orthotropic 3D continuous structure with interfacial bonding by a bottom-up technique (Fig. 1b). Frist, high-quality GO was controllably assembled into a GOF. After surface-functionalization modification and high-temperature pyrolysis of the GOF, SiO_2_-HOGF was formed. The VACNTs were synthesized in situ on the SiO_2_-HOGF surface via FCCVD and SiO_2_ was transformed to SiC in situ by an annealing treatment. Considering the prerequisites of interfacial heat-transfer applications, PDMS was chosen as a matrix material with intrinsic flexibility and adhesion. The PDMS was fully formed and cured in VSCG to obtain the VSCG/PDMS composite. Because the VSCG is hierarchically ordered (3D, orthotropic, and continuous) and PDMS is mechanically adaptable, the self-standing VSCG/PDMS is customizable, self-adhesive, and deformable (Fig. S1), besides conferring high thermal conductivity in 3D space.

The framework structure (such as the densification degree, orientation, and interface) critically affects the mechanical and thermal properties of the composites. Therefore, a series of VSCG networks (VSCG-0.01, VSCG-0.03, and VSCG-0.1) with different microstructures was fabricated by controlling the catalyst (ferrocene) concentration (0.01, 0.03, and 0.1 g mL^−1^, respectively) in the FCCVD process. The atopic structures and degrees of orientation (order parameters *f*) of the VSCGs were characterized using scanning electron microscopy (SEM) and small angle X-ray scattering (SAXS), respectively. The HOGF and VACNTs endowed all VSCG networks with a similar macroscopic structure (Fig. 1c–e). All networks present a long-range continuous orthotropic framework attributed to the robust bonding structure of the HOGF/VACNTs interface, as demonstrated in previous work [24]. However, the distributions and orientations differ among the VSCG-0.01, VSCG-0.03, and VSCG-0.1 networks. The VSCG-0.01 network presents a uniform and smooth surface (Fig. S2a) and freer vertical alignment of the internal CNTs than the other networks (Fig. 1c, inset). The VSCG-0.03 network prepared with a higher catalyst concentration displays a surface with microcracks (Fig. S2b) and the internal CNTs form bundles of spiral arrays (Fig. 1d, inset). In the VSCG-0.1 network prepared with the highest catalyst concentration, the surface microcracks are expanded from those of the VSCG-0.03 network (Fig. S2c) and the internal CNTs are entangled to form a 3D cross-linked structure (Fig. 1e, inset). The elliptical SAXS patterns of the VACNTs within the three samples typify an anisotropic structure (Fig. 1c–e, inset). Notably, the anisotropy of the VACNTs gradually weakens with the catalyst concentration. From the background-corrected azimuthal-intensity scan function curves in the 2D dataset, the *f* values of VACG-0.01, VSCG-0.03, and VSCG-0.1 network were calculated as 0.85, 0.74, and 0.66, respectively, further indicating that the vertical alignment behavior of the VACNTs within the samples deteriorates with increasing catalyst concentration (Fig. S3). This phenomenon can be explained by the numerous catalytic sites on the SiO_2_-HOGF surface and CNTs after pyrolysis of a sample with high catalyst concentration under high-temperature conditions. These sites cause entanglement of neighboring CNTs during the growth process, thereby decreasing the vertical orientation of the VACNTs [25, 26]. Nevertheless, high-density catalytic sites facilitate the CNT growth and improve the densification degree of the VSCG network. As demonstrated in the above results, the simple FCCVD strategy realized the continuous regulation of VACNTs on the HOGF surface, from a low-density vertically oriented array structure to a high-density 3D cross-linked network structure. Therefore, this strategy provides abundant and promising carbon-based hybrid thermally conductive frameworks for polymer matrices.

A previous study found that many conventional polymers can solubilize or homogeneously encapsulate CNTs or graphene through non-specific interactions [27]. Fourier transform infrared spectroscopy (FTIR) identified a C–H⋯*π* interaction between the hydrocarbon group (–CH) on the polymer chain and the highly dispersed large *π*-bonds on CNT or graphene surfaces [28, 29]. In the present study, the frequency, intensity, and shape evolution of the C–H vibrational peak in the FTIR spectra of the VSCG/PDMS composites and pure PDMS confirmed a similar C–H⋯*π* interaction between VSCG and PDMS (Fig. S4). This result clarifies that PDMS easily filled the pores of VSCG during vacuum-assisted impregnation, forming dense cross-sectional structures with high interfacial compatibility (VSCG-0.01/PDMS, VSCG-0.03/PDMS and VSCG-0.1/PDMS; see Fig. 1f–h). Meanwhile, PDMS formed a continuous coating on the surface of each VSCG network (Fig. S5). The network structure of VSCG remained largely intact in the composites, which is essential for improving the comprehensive mechanical and thermal properties.

### Thermally Conductive Performance of VSCG/PDMS Composites

**Fig. 2:**
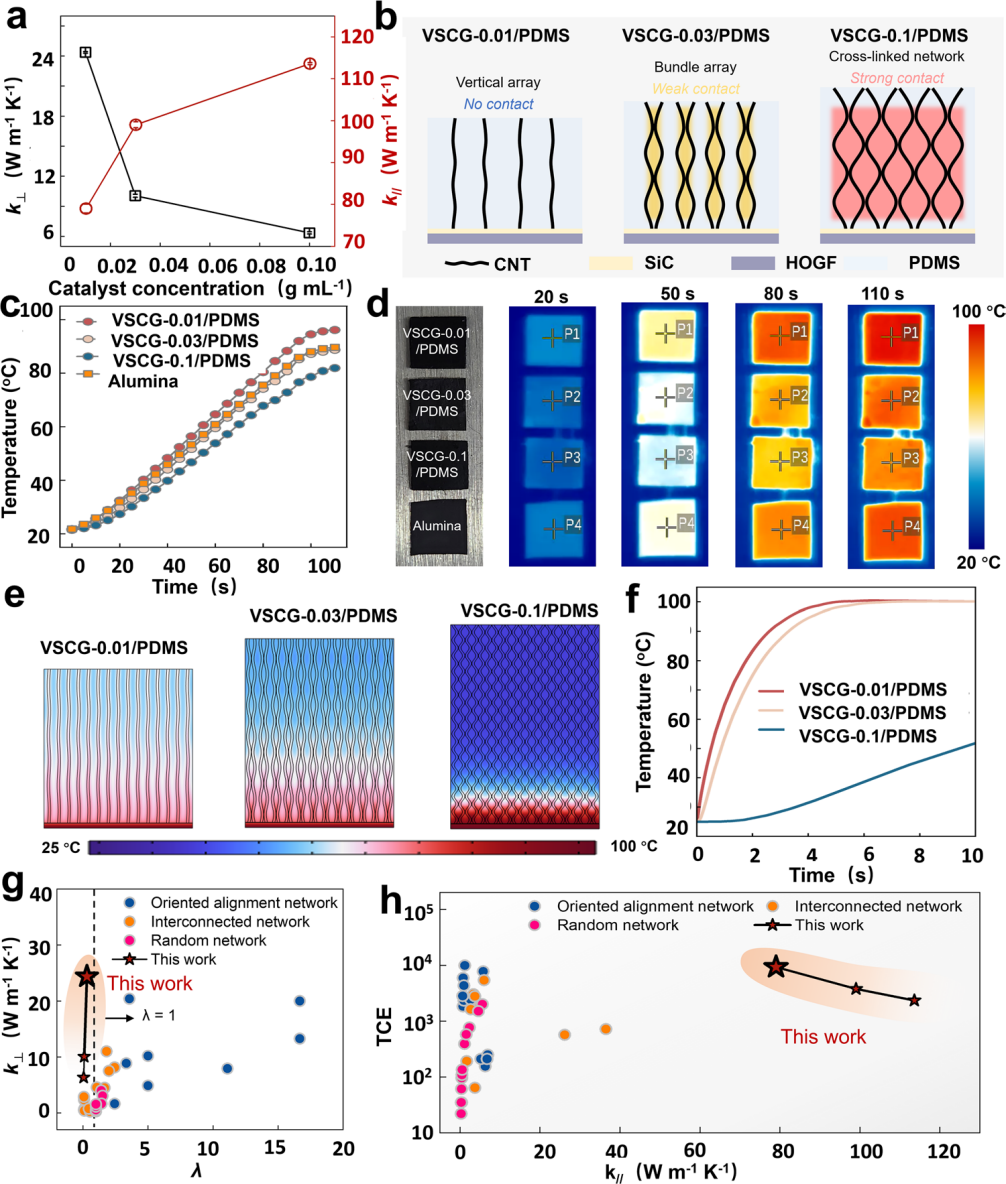
**a** In-plane and through-plane conductivities (*k*_*∥*_ and *k*_⊥_, respectively) of the VSCG/PDMS composites vs. catalyst concentration; **b** structural evolution of the VSCG/PDMS composites; **c** temperature evolutions of the VSCG/PDMS composites; **d** corresponding IR images of the VSCG/PMDS composites during thermal conduction along the through-plane direction; **e** simulated comparison of thermally conductive capacities of the VSCG/PDMS composites along the through-plane direction and **f** the corresponding surface-temperature evolutions; comparisons of **g** *k*_⊥_ vs. *λ* and **h** TCE vs. *k*_*∥*_ correlations in the VSCG/PDMS and previously reported composites with different types of networks (*λ* = thermally conductive anisotropy coefficient; TCE = thermal conductivity enhancement)

The orthotropically continuous VSCG networks provide 3D ordered phonon conductive pathways. Thus, the thermally conductive property of VSCG/PDMS composites greatly depends on the densification degree and orientation structure in the VSCG networks. Because the HOGFs were prepared under the same conditions when fabricating the VSCG networks, the network microstructures were mainly influenced by the growth pattern of the VACNTs, i.e., the catalyst concentration. When the catalyst concentration increased from 0.01 to 0.1 g mL^−1^, the VSCG network density enlarged from 0.71 to 0.83 g cm^−3^ (Fig. S6). After compounding, the densities of the corresponding VSCG/PDMS composites with 0.01, 0.3, and 0.1 g mL^−1^ catalyst were further enlarged to 0.89, 0.95, and 0.98 g cm^−3^. The increased VACNT density improved the densification degree of the VSCG networks and enhanced the *π*–*π* interactions between adjacent CNTs. Consequently, many continuous thermal conductive pathways formed in the horizontal direction and the phonon conductive efficiency was enhanced along the in-plane direction (Fig. 2a). As the VACNTs evolved from the free vertical alignment state through the bundled state to the 3D cross-linked structure, the in-plane thermal diffusivities (*α*_*∥*_) in the corresponding VSCG/PDMS composites increased from 67.26 (VSCG-0.01/PDMS) to 98.34 (VSCG-0.03/PDMS) to 115.93 (VSCG-0.1/PDMS) mm^2^ s^−1^ (Table S1). In contrast, the through-plane thermal diffusivity (*α*_⊥_) decreased from 20.74 (VSCG-0.01/PDMS) to 9.98 (VSCG-0.03/PDMS) to 6.47 (VSCG-0.1/PDMS) mm^2^ s^−1^ (Table S1). This trend derives from the reduction in the vertically ordered degree of VACNTs (decreasing *f* from 0.85 to 0.66) and the increased number of CNT–SiC–graphene nodes as scattering centers (Fig. 2b). Owing to the atopic structure of the VSCG network, VSCG-0.01, VSCG-0.03 and VSCG-0.1 achieved in-plane thermal conductivities (*k*_*∥*_) of 79.02, 99.03, and 113.61 W m^−1^ K^−1^, respectively, and through-plane thermal conductivities (*k*_⊥_) of 6.34, 10.05, and 24.37 W m^−1^ K^−1^, respectively (Fig. 2a).

To verify the excellent thermal conductivity of the VSCG/PDMS composites, the thermal conductive capacities of the VSCG/PDMS composites were compared with that of stainless steel. Prior to testing, the three VSCG/PDMS composites and stainless steel were cut to the same lateral size of 10 × 10 mm^2^ and sprayed with a graphite coating. The samples were simultaneously heated on a heating stage and their surface-temperature evolutions were recorded with an infrared thermal imager. The graphite coating excluded interference caused by the different infrared emissivities of the four samples during the infrared temperature measurements. The VSCG-0.01/PDMS exhibited the highest thermally conductive rate and the highest steady-state surface temperature among the samples, including stainless steel (Fig. 2c, d). Judging from this result, the highly vertically ordered VACNTs and horizontally aligned HOGF synergistically provide the preferred phonon conduction pathways, thus enhancing the comprehensive thermal conductive performance along the through-plane direction.

Carbon nanotubes are grown on the surface of graphene, the carbon tubes and graphene provide continuous thermal conductivity to improve the effective transmission efficiency of phonons. The continuous interfacial bonding between graphene and carbon material effectively reduces the thermal resistance and improves the thermal conductivity of the composite material [30]. To theoretically understand the enhanced thermally conductive efficiency of the three VSCG networks (vertical array, bundled array, and cross-linked network) in the PDMS matrix, the thermally conductive capacities of the VSCG/PDMS composites were compared in a simulation based on finite-element analysis (FEA) and executed in computational fluid dynamics software (COMSOL). The bottom of each model was supplied with a constant temperature load of 100 °C to generate a unidirectional heat flow (Fig. 2e). Among the three models, the VSCG-0.01/PDMS model presented the highest top temperature and reached steady state within the shortest time (Fig. 2f), consistent with the above test results. However, at a given position from the bottom, the VSCG-0.1/PDMS composite achieved a more uniform temperature distribution than the VSCG-0.01/PDMS and VSCG-0.03/PDMS composites (Fig. S7). This result was attributed to the anisotropic thermal conductivity within the orthotropically continuous VSCG network of VSCG-0.1/PDMS. Next, the thermally conductive behaviors of the VSCG/PDMS composites were quantified in terms of their thermally conductive anisotropy coefficients (*λ* = *k*_*∥*_/*k*_⊥_) [31]. Here, *λ* = 1 indicates completely isotropic thermal conduction, whereas near-zero *λ* values indicate unidirectional thermal conduction. Reducing the *λ* of TIMs is essential for raising the *k*_⊥_, thereby achieving rapid thermal conduction along the vertical direction while enhancing the homogeneity of heat distribution within the materials. The *λ* of the VSCG-0.01/PDMS, VSCG-0.03/PDMS, and VSCG-0.03/PDMS composites reached 0.31, 0.10, and 0.06, respectively (Fig. 2g and Table S2), comparable to those in most reported random and interconnected networks but substantially improved from those of oriented alignment networks. Meanwhile, the *k*_⊥_ values of the composites were far superior to those of random and interconnected networks and equivalent to those of oriented alignment networks. Figure 2h plots the thermal conductivity enhancement (TCE) of the VSCG/PDMS composites along the through-plane direction as a function of *k*_*∥*_. The TCE, defined as (*k*_⊥_ − *k*_0_) × 100%/*k*_0_ (*k*_0_ ≈ 0.26 W m^−1^ K^−1^) [32], reached a 10^4^ order of magnitude in the VSCG/PDMS composites, comparable to those of oriented alignment networks. However, the uniquely advantageous *k*_*∥*_ of the VSCG/PDMS composites is not easily matched by the other three network types.

### Mechanical Performance of VSCG/PDMS Composites

**Fig. 3:**
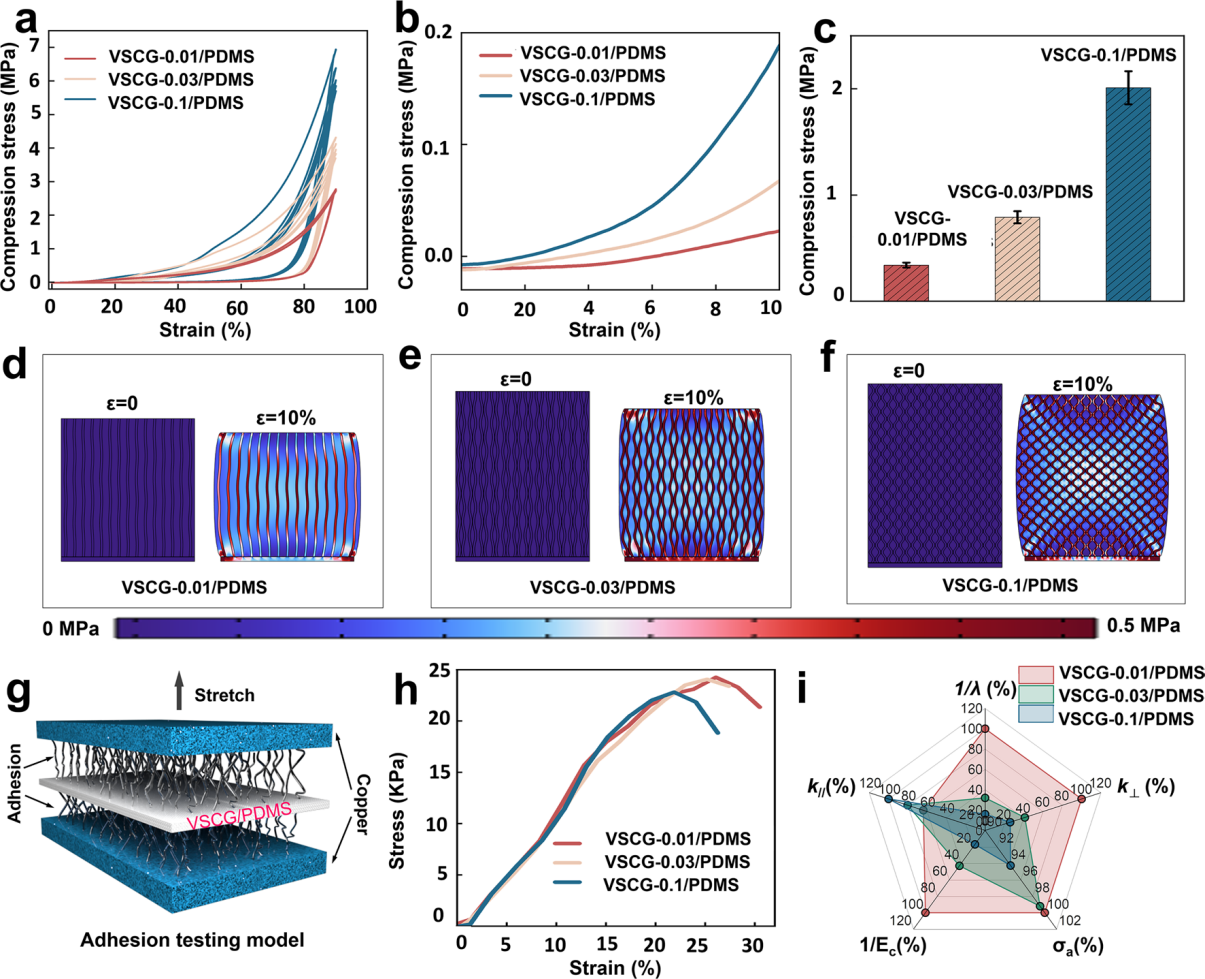
**a** Applied load vs. strain plots of the VSCG-0.01/PDMS, VSCG-0.03/PDMS, and VSCG-0.1/PDMS composites strained up to 90%; **b** enlarged view of **a** between 0 and 10% strain; **c** comparison of compression modulus of the VSCG/PDMS composites; simulated stress profiles of **d** VSCG-0.01/PDMS, **e** VSCG-0.03/PDMS and **f** VSCG-0.1/PDMS before and after 10% strain loading; **g** model for testing adhesion between the VSCG/PDMS composites and Cu metal; **h** the modeled adhesive strength–strain curves; **i** comparison of thermal conductivity, compressibility and adhesion properties of the VSCG/PDMS composites

For practical applications in TIMs, the VSCG/PDMS composites must possess both excellent thermally conductive ability and sufficient mechanical properties [33]. TIMs with high compression resilience can rapidly deform and easily maintain strong contact with the heater and the heat sink. Such abilities guarantee efficient interfacial heat-transfer in the thermal-management system. Figure 3a exhibits the stress–strain curves of the VSCG/PDMS composites during continuous compression cycles. When the strain increased to 90%, the stress in the VSCG-0.01/PDMS composite remained above 95% even after five compression cycles, indicating that this composite can effectively resist thermal stresses generated by heater vibrations. In contrast, the VSCG-0.03/PDMS composite and (more notably) the VSCG-0.1/PDMS composite were degraded during the compression cycles. Ultra-high strain probably caused slip or structural damage of the bundled CNTs within the VSCG-0.03 composite or the randomly entangled CNTs within the VSCG-0.1 composite, along with interface separation from the matrix [34]. Furthermore, the first-loop compressive stresses in the VSCG-0.01/PDMS, VSCG-0.03/PDMS, and VSCG-0.1/PDMS composites under 90% compressive strain were 2.77, 4.31, and 6.94 MPa, respectively. This increasing trend was attributed to the increased number of entangled CNTs under compression, which exerted a reinforcing effect [35]. Notably, all samples showed hysteretic return lines, indicating viscoelastic mechanical responses of their VSCG and PDMS under compression. This behavior is related to the stress relaxation that typifies compressible carbon networks and polymers. Figure 3b presents the stress–strain curves of the VSCG/PDMS composites in the 0–10% strain range. Under 10% strain, the compressive stresses of VSCG-0.01/PDMS, VSCG-0.03/PDMS, and VSCG-0.1/PDMS were 0.02, 0.07, and 0.19 MPa, respectively, well satisfying the low compressive-stress requirement of packaged TIMs [32]. However, as the stress–strain behaviors of the VSCG/PDMS composites were nonlinear in this range, their compressibilities were investigated in terms of the corresponding tangential modulus (*E*_c_ = d*σ*/d*ε*), where *σ* and *ε* are the compressive stress and strain, respectively (Fig. S8). The average *E*_c_ values representing the compression modulus of VSCG-0.01/PDMS, VSCG-0.03/PDMS, and VSCG-0.1/PDMS were 0.34, 0.79, and 2.01 MPa, respectively. Such good compressibility confers excellent flexibility and deformability, facilitating the filling of thermal interface gaps to connect the thermal pathways (Fig. 3c).

Filling the PDMS with the VSCG network can effectively improve the structural stability of the material, reduce the phonon scattering between the thermal conduction paths, and make the thermal conductivity of the VSCG/PDMS composites more effective [36, 37]. The contribution of the VSCG microstructure to the mechanical properties of the VSCG/PDMS composites were further investigated through a FEA using COMSOL software. The simulation parameters are given in Table S3 and the simulated stress distributions in the three VSCG/PDMS composites before and after compression under the same compressive load are plotted in Fig. 3d–f. Under compression, the dense cross-linked network in the VSCG-0.1/PDMS composite bundled the internal PDMS matrix, causing strong stress concentration at the CNT-SiC-graphene nodes and extrusion of the internal PDMS. This behavior enhanced the compression modulus but the expanded PDMS caused slippage at the matrix-network interface. In contrast, the stress was uniformly distributed through the PDMS filled in the low-density vertically oriented array of the VSCG-0.01/PDMS composite and was mainly concentrated at the CNTs in the VSCG-0.01 network. Accordingly, the VSCG-0.01/PDMS composite was freely compressible and demonstrated high compression-cycling performance.

The PDMS also conferred a self-adhesive property to the VSCG/PDMS composites. When sandwiched between two copper plates, the VSCG-0.01/PDMS, VSCG-0.03/PDMS and VSCG-0.1/PDMS composites achieved adhesive strengths *σ*_a_ of 24.26, 24.07, and 22.84 kPa, respectively (Fig. 3g, h). This decreasing trend is possibly attributed to roughening of the VSCG tip with increasing catalyst concentration, which decreases the true contact area between VSCG/PDMS and copper (Cu), thus lessening the adhesion at the contact interface. Nonetheless, owing to the self-adhesive capacity, the contact between the VSCG/PDMS composites and their matching surfaces is much more stable than that of conventional TIMs (which provide only non-adhesive interface bonding) during long-term utilization. Such property also avoids expansion separation at the heater–TIM–heat-sink interface under dense heat flows. Through this comprehensive comparison of the thermal conductive and mechanical properties of the VSCG/PDMS composites (Fig. 3i), the VSCG-0.01/PDMS composite with the lowest *λ* and compression modulus and highest *k*_⊥_ and *σ*_a_ emerged as the preferred TIM. This composite is expected to improve the efficiency of heat transfer across the interface from the heater to the heat sink, thus improving the cooling efficiency of a thermal-management system.

### Thermal Management Performance of VSCG/PDMS Composites

**Fig. 4:**
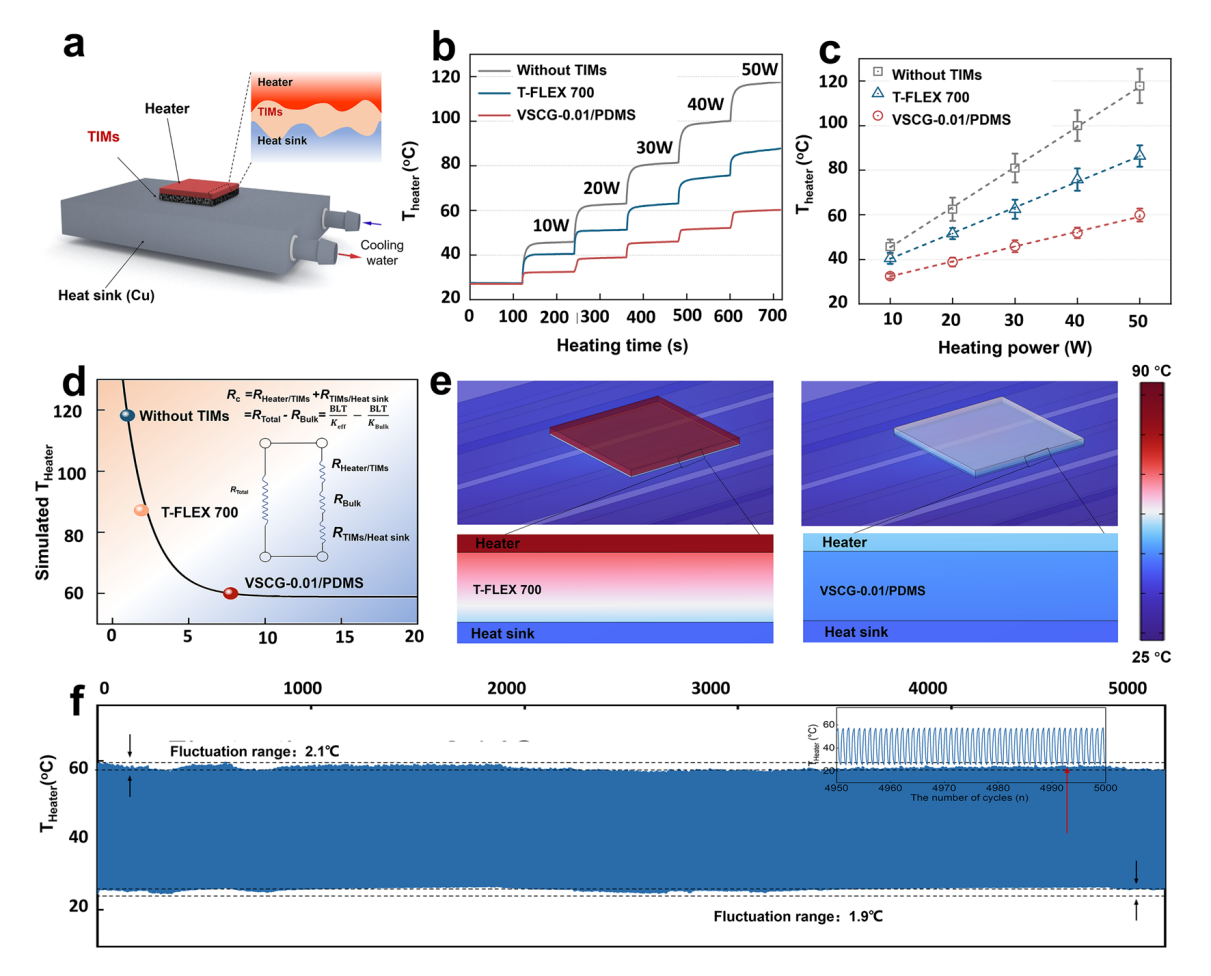
**a** Schematic showing the interfacial heat-transfer properties of TIMs; **b** temporal surface-temperature evolution of the heater at different powers; **c** surface steady-state temperatures of the heater as functions of power in the VSCG-0.01/PDMS and T-FLEX 700 TIMs; **d** simulated *T*_Heater_ vs. *k*_eff_ of TIMs; **e** simulated temperature profiles of cooling systems integrated with VSCG-0.01/PDMS and T-FLEX 700 TIMs; **f** thermal shock stability during cyclic heating/cooling tests of the VSCG-0.01/PDMS TIM

To evaluate the interfacial heat-transfer performances of the VSCG-0.1/PDMS composite as a TIM, the composite was packed between a ceramic heater and a Cu heat sink, forming a common cooling system. The heat generated after turning on the heater was conducted along the TIM to the heat sink and finally cooled at the water-cooled terminal (Fig. 4a). During this process, the real-time temperature evolution at the heater surface (*T*_Heater_) was recorded with a thermocouple. For comparison, a commercial silicon-based thermal pad (T-FLEX 700: 5.00 W m^−1^ K^−1^; Laird, USA) was tested at the same bond line thickness (BLT ≈ 310 μm). As the heater power increased, the system with the VSCG-0.01/PDMS composite achieved higher interfacial heat-transfer efficiency (i.e., lower *T*_Heater_) than the systems with commercial T-FLEX 700 and without a TIM (Fig. 4b). At a heater power of 50 W, the temperature drop of the heater was considerably higher in the VSCG-0.01/PDMS-integrated system than in the T-FLEX 700-integrated system (57.9 vs. 31.4 °C from that of the steady-state *T*_Heater_ without a TIM). The cooling efficiency of the system was evaluated by linearly fitting the plot of steady-state *T*_Heater_ vs. heating power. The equivalent heat-transfer coefficients (calculated as the reciprocal of the slope) were 0.56, 0.87, and 1.49 W °C^−1^ in the without a TIM, T-FLEX 700, and VSCG-0.01/PDMS systems, respectively (Fig. 4c). That is, VSCG-0.01/PDMS enhanced the interfacial heat-transfer efficiency by 71.3% and 166.1% from those of the without a TIM and T-FLEX 700 systems, respectively.

Next, the heat-dissipation process of the cooling system at a heater power of 50 W was analyzed through a steady-state FEA. The simulation model and corresponding parameters are shown in Fig. S9a–c and Table S4, respectively. As seen in Fig. 4d correlating the simulated *T*_Heater_ and the effective thermal conductivity (*k*_eff_), the *k*_eff_ majorly determines the cooling efficiency of the system. The calculated *k*_eff_ of the VSCG-0.01/PDMS composite was 7.75 W m^−1^ K^−1^, more than four times that of T-FLEX 700 (1.90 W m^−1^ K^−1^). Accordingly, the *T*_Heater_ of the VSCG-0.01/PDMS-integrated system (60.0 °C) was reduced by 27.2 °C from that of the T-FLEX 700-integrated system (87.2 °C). The contact thermal resistance was calculated as *R*_c_ = *R*_Heater/TIMs_ + *R*_TIMs/Heat sink_ = *R*_Total_ − *R*_Bulk_ = BLT/*k*_eff_—BLT/*k*_Bulk_ (where *R*_Heater/TIMs_, *R*_TIMs/Heat sink_, *R*_Total_, *R*_Bulk_, and *k*_Bulk_ denote the contact thermal resistance of a TIM with a heater and heat sink, the total thermal resistance of the system, the intrinsic thermal resistance of the material, and the thermal conductivity of the materials, respectively). The *R*_c_ was much lower in the VSCG-0.01/PDMS-integrated system (26.40 K mm^2^ W^−1^) than in the T-FLEX 700-integrated system (97.89 K mm^2^ W^−1^; see Table S5). This result derived from the high *k*_⊥_, large self-adhesion, and lower compression modulus of VSCG-0.01/PDMS than of T-FLEX 700 (0.32 MPa vs. 0.38 MPa; Fig. S10). As shown in the simulated results (Figs. 4e and S9d–f), the cooling system integrated with the VSCG-0.01/PDMS composite exhibited a lower and more uniform temperature profile than the T-FLEX 700-integrated system, visually demonstrating that VSCG-0.01/PDMS improves the efficiency of interfacial heat transfer and raises the vertical heat flux. Furthermore, the heater was continuously switched on and off (50/0 W) to simulate the thermal shock condition during real operation of TIMs. The temperature fluctuations of the heater were small, being maximized at 2.1 °C and minimized at 1.9 °C during fast and long-term variable heat-flow environments, respectively (Fig. 4f). Therefore, when employed as a TIM, the VSCG-0.01/PDMS composite provides excellent thermal shock resistance and does not degrade the bonding interface.

**Fig. 5:**
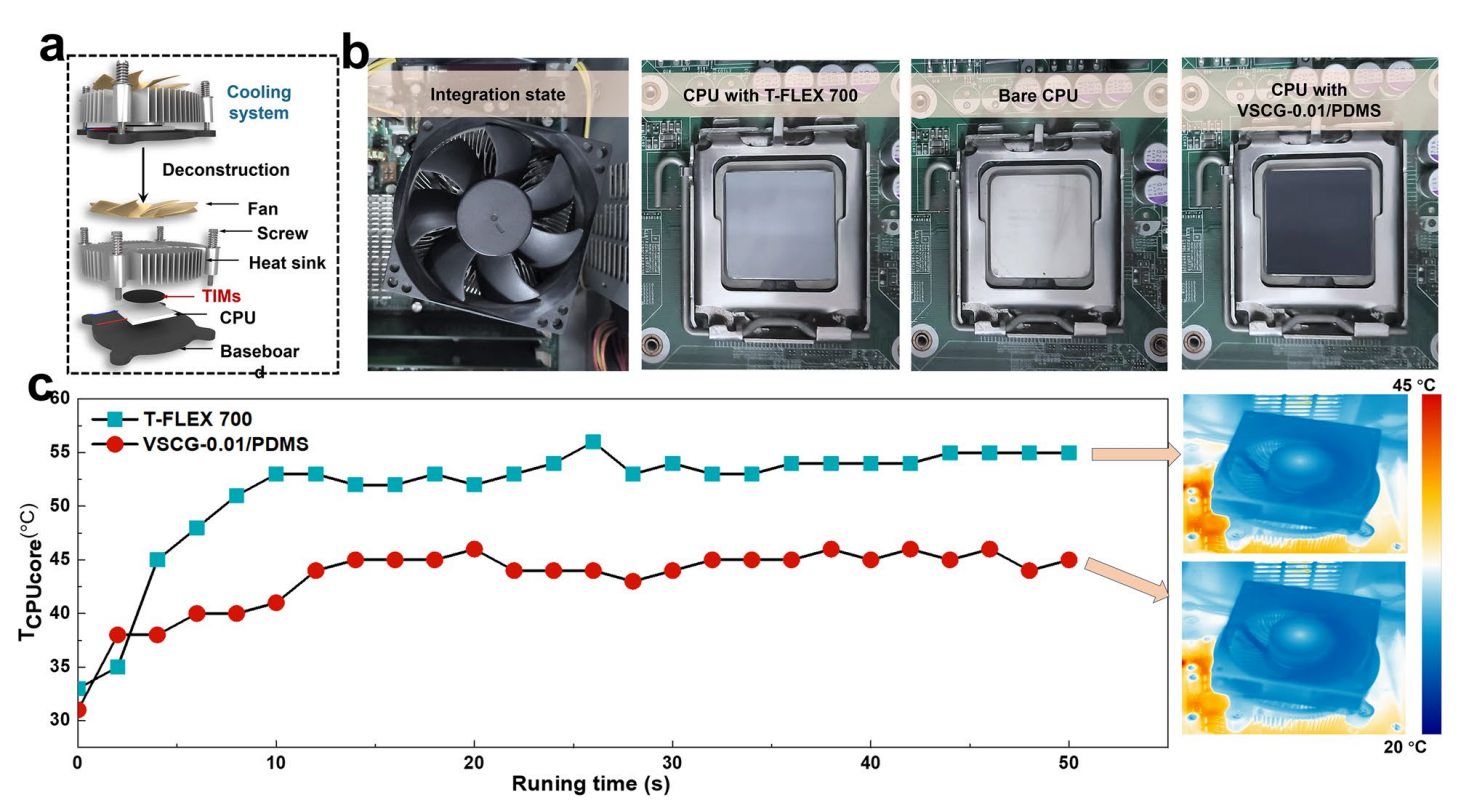
**a** Experimental setup and **b** photographs for comparing the actual thermal-management performances of the VSCG-0.01/PDMS and T-FLEX 700 TIMs; **c** temperature evolutions of a CPU core during full load operation (plots) and IR images inside the chassis

To demonstrate the thermal-management performance of the VSCG-0.01/PDMS composite as a TIM in practical applications, the composite was installed on a platform for cooling the central processing unit (CPU) of a computer. A schematic and photographs of the test setup are shown in Fig. 5a, b, respectively. Before testing, the VSCG-0.01/PDMS composite and T-FLEX 700 TIMs were placed between the CPU and an aluminum heat sink and the heat was eventually carried away through the forced air-cooling terminal. Subsequently, the CPU was operated at full load and its evolving core temperature (*T*_CPU core_) was recorded using professional on-demand software (Core Temp). When integrated with the VSCG-0.01/PDMS composite, the CPU reached steady state faster than when integrated with T-FLEX 700 (Fig. 5c) and its steady-state *T*_CPU core_ at 50 s was 12.0 °C lower than when integrated with T-FLEX 700. As clarified in the IR images, the chassis integrated with the VSCG-0.01/PDMS composite was well cooled and its temperature distribution was relatively uniform. In contrast, heat accumulation was evident in the chassis integrated with T-FLEX 700. The above results further demonstrate that owing to its superior thermal-management performance, the VSCG-0.01/PDMS composite facilitates the efficient operation of devices. Consequently, the VSCG-0.01/PDMS composite can be expected as a new type of TIM that can replace commercial silicon-based TIMs. Especially, the composite meets the heat-dissipation requirements of new-generation electronic devices, which are highly integrated with high-power density. It also provides many options for efficient thermal-management designs.

## Conclusions

A class of orthotropic 3D hybrid carbon networks (VSCG) with controlled microstructure was fabricated by regulating the FCCVD process of VACNTs on a HOGF surface, followed by in situ annealing. As the catalyst concentration increased in the FCCVD process, the obtained VACNTs within the VSCG network evolved from a low-density vertically oriented array structure to a high-density 3D cross-linked network. Owing to the rational surface-interface structural design of the VSCG and its strong interfacial compatibility with PDMS, the VSCG/PDMS composite achieves high compression resilience (90% strain resilience), compressibility (compression modulus = 0.34 MPa), strong adhesion, and a high 3D thermal conductivity (in-plane: 113.61 W m^−1^ K^−1^; through-plane: 24.37 W m^−1^ K^−1^). During the TIM performance evaluation, the interfacial heat-transfer efficiency of the VSCG/PDMS composite was 71.3% higher than that of a commercial thermal pad T-FLEX 700 (≈ 5 W m^−1^ K^−1^). This performance was attributed to the excellent through-plane thermal conductivity and efficient spreading of heat from hot spots through the VSCG network. The finite-element simulations demonstrated that lowering the compression modulus reduces the contact thermal resistance. When the VSCG/PDMS composite was applied as a TIM for actual thermal-management of a computer CPU, it lowered the steady-state temperature by 12.0 °C from that of the T-FLEX 700 pad. Besides providing a new perspective for balancing high thermal conductivity with low contact thermal resistance, this study suggests an effective replacement of commercial silicon-based TIMs.

## Supplementary Information

Below is the link to the electronic supplementary material.

Supplementary file1 (PDF 1082 KB)
