# Supplementary material for: Regulatable Orthotropic 3D Hybrid Continuous Carbon Networks for Efficient Bi-Directional Thermal Conduction
Source: Nanomicro Lett. 2024 May 17;16:198. doi: 10.1007/s40820-024-01426-0 (PMC11101387; doi:10.1007/s40820-024-01426-0)
Supplement: Supplementary file 1 — Supplementary file1 (PDF 1082 KB) [file 40820_2024_1426_MOESM1_ESM.pdf]

Supporting Information for

Regulatable Orthotropic 3D Hybrid Continuous Carbon Networks for Efficient Bi-Directional Thermal Conduction

Huitao Yu[1], Lianqiang Peng[1], Can Chen[1], Mengmeng Qin[1,*], and Wei Feng[1,*]

[1] School of Materials Science and Engineering and Tianjin Key Laboratory of Composite and Functional Materials, Tianjin University, Tianjin 300350, P. R. China

*Corresponding authors. E-mail: weifeng@tju.edu.cn (Wei Feng); qmm@tju.edu.cn (Mengmeng Qin)

S1 Structural Analysis and Characterization

The morphologies of the materials were evaluated using field-emission scanning electron microscopy (SEM; Sigma 300, ZEISS, Germany). Fourier transform infrared spectra (FTIR) was recorded by ALPHA II (Bruker, USA). Small-angle X-ray scattering (SAXS) experiment was performed using a two-dimensional SAXS instrument (Xeuss 2.0, Xenocs, France), and the sample-to-detector distance was fixed at 2490 mm. Order parameter (f) of the representative crystalline lattice is defined as follows [S1]:

$$f = \frac{3<cos^2\varphi>-1}{2}$$

$$cos^2\varphi = \frac{\int_0^{\frac{\pi}{2}} I(\varphi)\, cos^2\varphi\, sin\varphi\, d\varphi}{\int_0^{\frac{\pi}{2}} I(\varphi)\, sin\varphi\, d\varphi}$$

where φ denotes the angle between the normal direction of the crystalline lattice and the reference axis (equatorial direction). The thermal diffusivity (α) was measured using a laser flash apparatus (LFA 467, Netzsch, Germany), the specific heat capacity (C_p) was measured using a differential scanning calorimeter (DSC) (Q20, TA Instruments, USA), and the density (ρ) was determined using the water displacement method. Surface temperature evolution and thermal images were captured using an infrared thermal imager (Tix640, Fluke, USA). Mechanical tests were performed on an electronic universal testing machine (UTM2) at a rate of 2 mm min^{-1}.

S2 Supplementary Figures and Tables

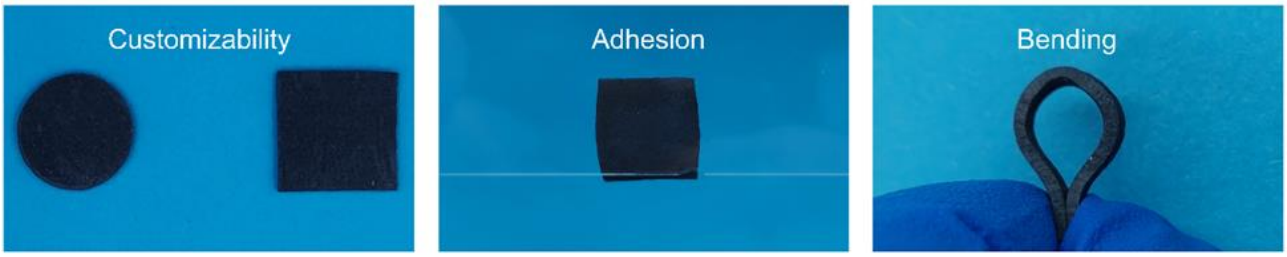

Fig. S1 The fabricated composite film is easily customizable, self-adhesive, and bendable

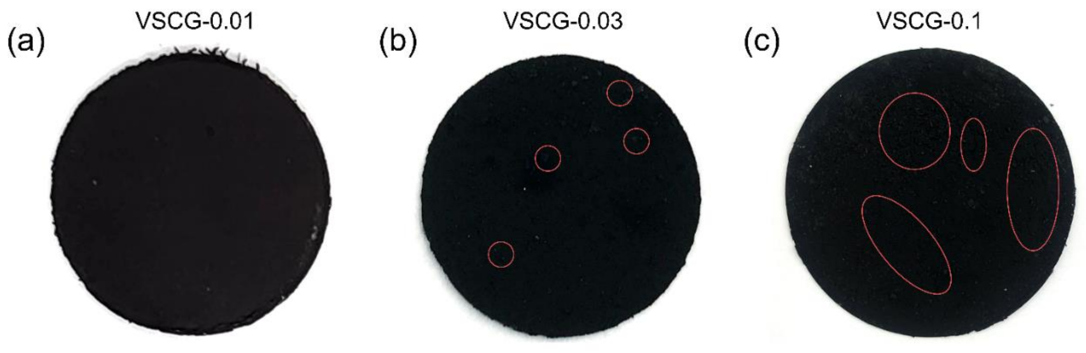

Fig. S2 Optical photograph of the (**a**) VSCG-0.01, (**b**) VSCG-0.03, and (**c**) VSCG-0.1 network

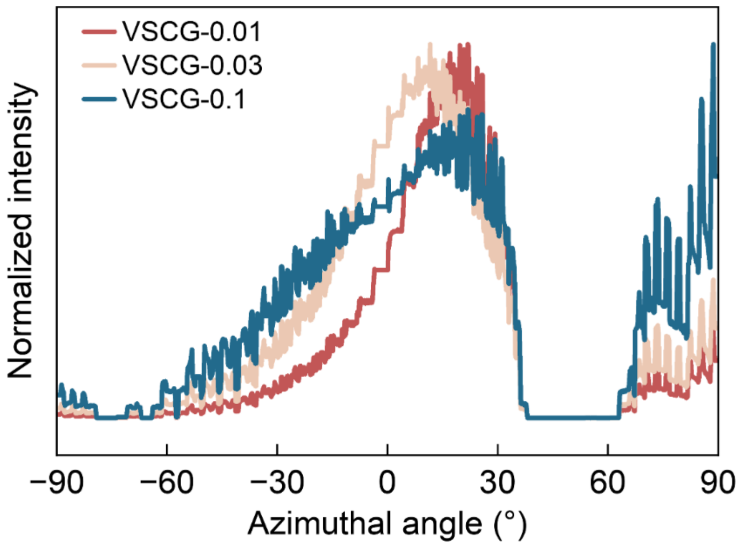

Fig. S3 Azimuthal angle plots from 2D SAXS diffraction patterns

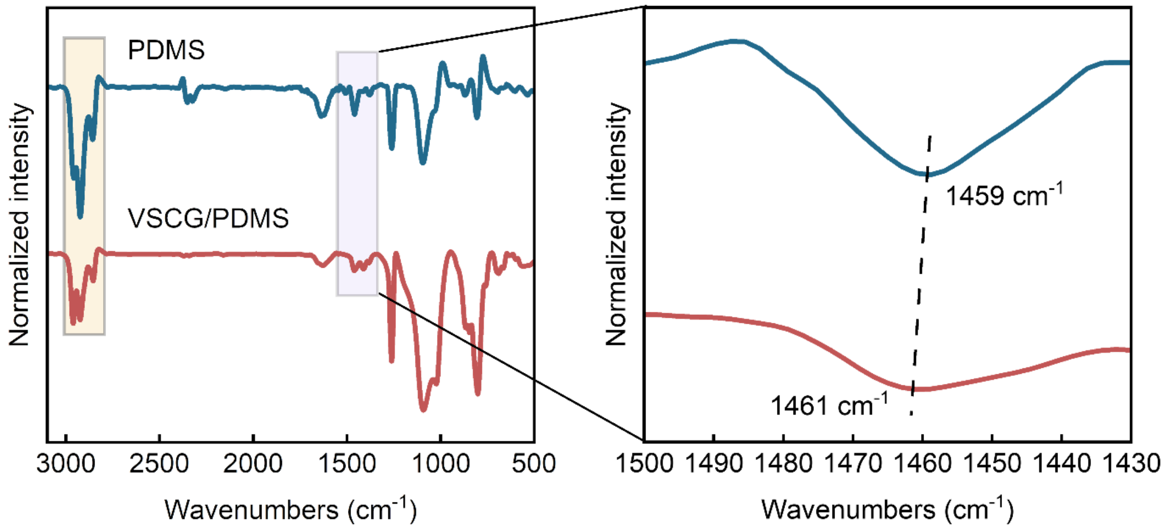

Fig. S4 FTIR of PDMS matrix and VSCG/PDMS composite

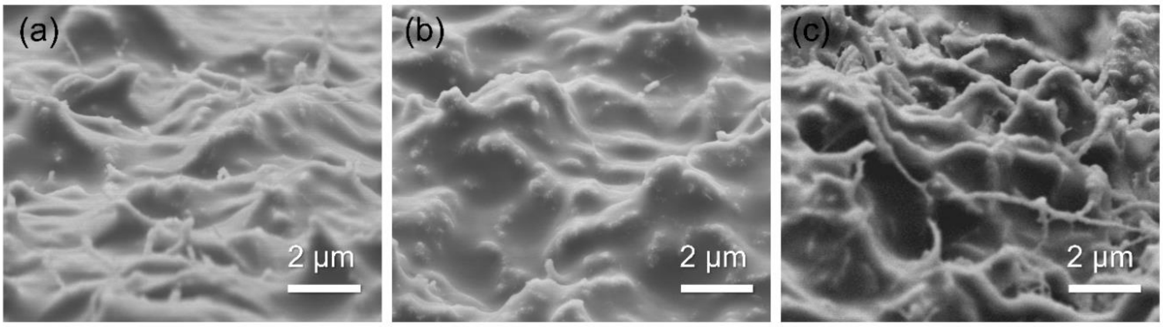

Fig. S5 SEM image of the surface of the (a) VSCG-0.01/PDMS, (b) VSCG-0.03/PDMS, and (c) VSCG-0.1/PDMS composite

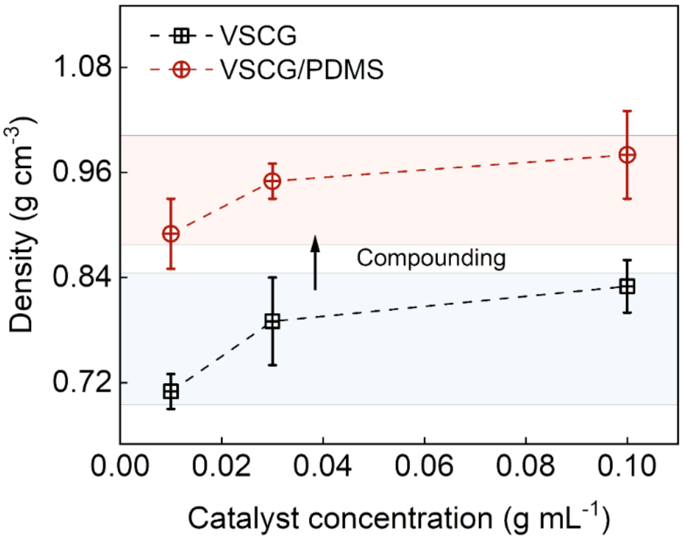

Fig. S6 Density of the VSCG networks and the VSCG/PDMS composites as a function of the catalyst concentration

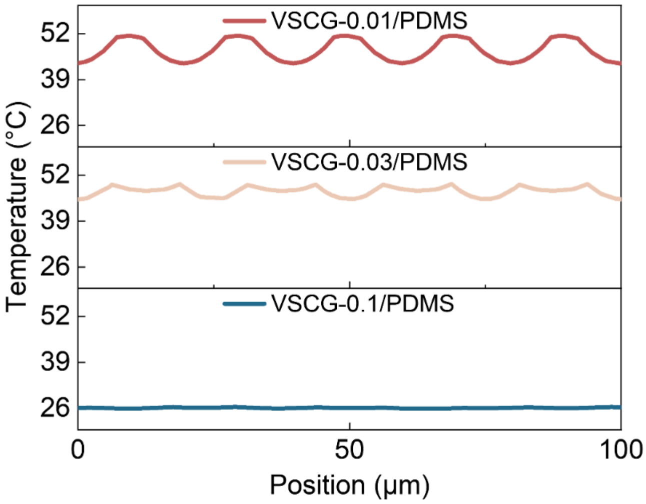

Fig. S7 Temperature profiles of different models at the same position from their bottom

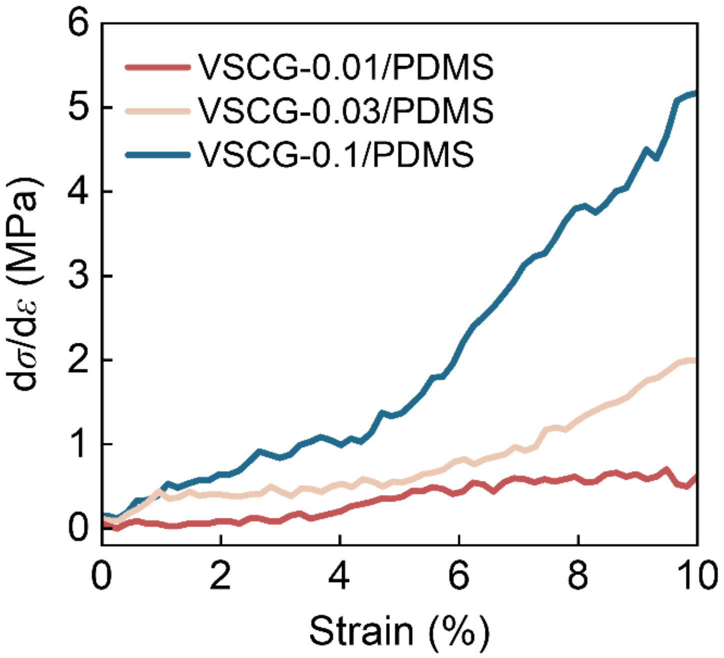

Fig. S8 dσ/dε of different VSCG/PDMS composites as a function of the strain

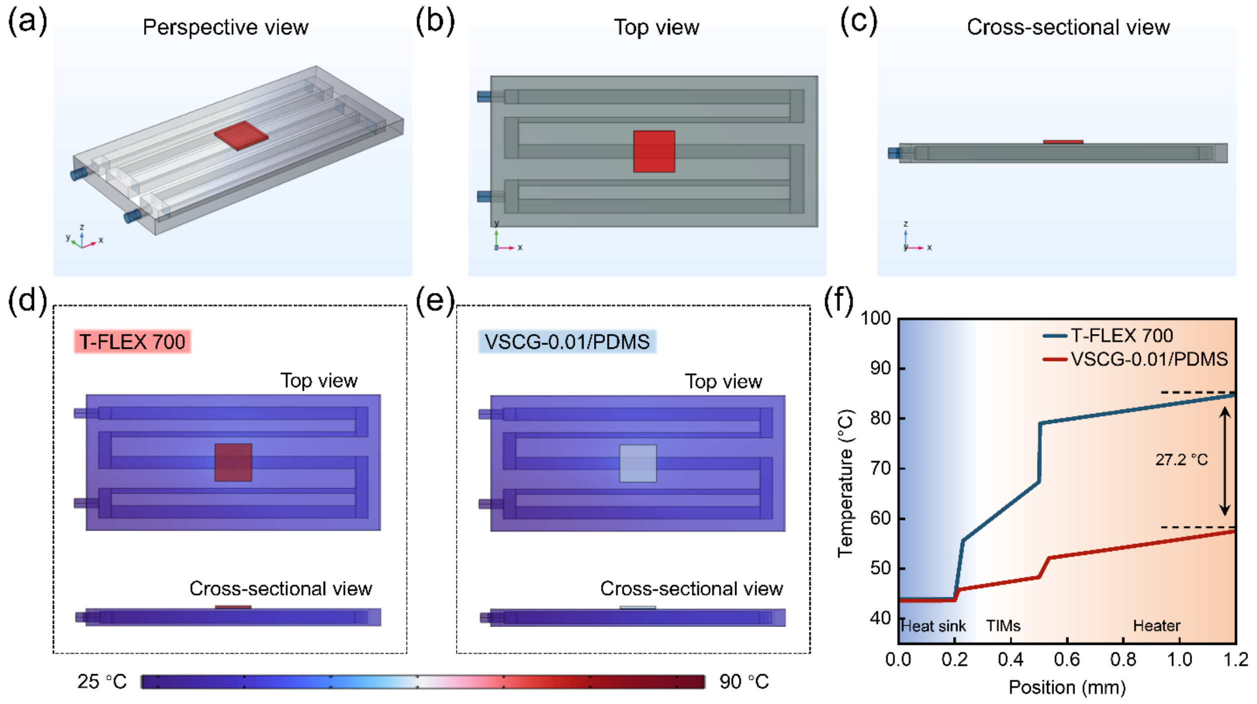

Fig. S9 (**a**) The perspective, (**b**) top and (**c**) cross-sectional view of Comsol model for TIM performance evaluation. The temperature distribution of the simulated cooling system integrated with (**d**) T-FLEX 700 and (**e**) VSCG-0.01/PDMS. (**f**) Temperature profiles from the bottom to the top center of the simulated cooling systems

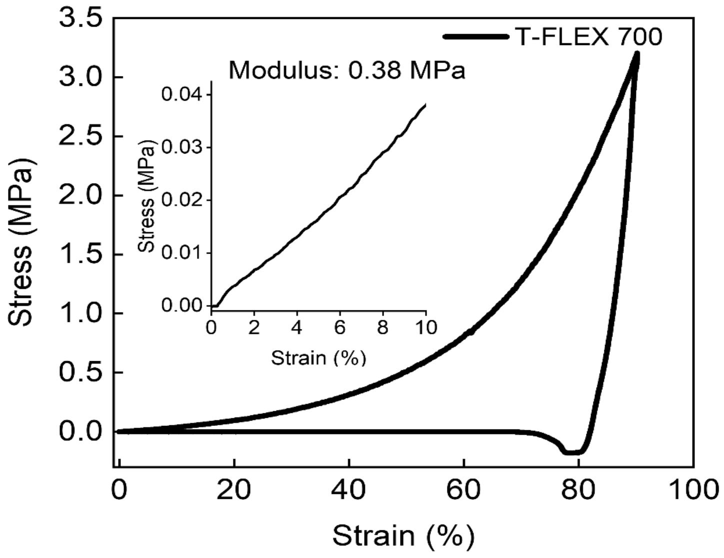

Fig. S10 The compressive stress-train curve of the T-FLEX 700

Table S1 The parameters for thermal conductivity of VSCG/PDMS composites as a function of the catalyst concentration in **Fig.** 3a

Sample	ρ (g cm^{-3})	Cp (J g^{-1} K^{-1})	$\alpha_{//}$ (mm^2 s^{-1})	$k_{//}$ (W m^{-1} K^{-1})	$\alpha_{\perp}$ (mm^2 s^{-1})	$k_{\perp}$ (W m^{-1} K^{-1})
VSCG-0.01/PDMS	≈0.89	≈1.32	67.26	79.02	20.74	24.37
VSCG-0.03/PDMS	≈0.95	≈1.06	98.34	99.03	9.98	10.05
VSCG-0.1/PDMS	≈0.98	≈1.00	115.93	113.61	6.47	6.34

Table S2 Comparison of thermal conductivity anisotropy (λ) and enhancement of VSCG/PDMS composites and other reported composites with different types of networks in **Fig.** 3g, h

Type	Filler	Matrix	TC of Matrix (W m^{-1} K^{-1})	$k_{\perp}$ of composites (W m^{-1} K^{-1})	$k_{//}$ of composites (W m^{-1} K^{-1})	λ	TCE$_{\perp}$ (%)	Refs.
Oriented alignment network	VACNTs	PANI	0.41	7.93	0.75	10.57	1834	[S2]
	Aligned CNTs	Epoxy	0.20	4.87	0.97	5.02	2335	[S3]
	GNWs	PDMS	0.26	20.40	5.80	3.52	7746	[S4]
	Natural graphite	POE	0.22	13.27	0.78	17.01	5931	[S5]
	Graphene nanoflake	PVDF	0.23	10.19	0.97	10.51	4330	[S6]
	Graphene aerogel	Epoxy	0.20	20.00	1.20	16.67	9900	[S7]
	Aligned GO	Paraffin	0.35	8.87	2.68	3.31	2434	[S8]
	CF	Epoxy	0.20	0.51	6.32	0.08	155	[S9]
				0.62	5.22	0.12	210	
				0.70	6.94	0.10	250	
				0.62	6.78	0.09	210	
	Graphene/Mxene	PEG	0.43	1.64	0.67	2.45	2814	[S10]
	BNNS	W-SPU		5.8	30.00	0.19	30–32	[S11]
	GWFs	PI	0.25	0.41	3.73	0.11	64	[S12]
	Graphite fiber	Paraffin	0.35	2.34	26.18	0.09	569	[S13]
				2.87	36.49	0.08	720	
	Graphene/CNT	PDMS	0.17	0.23	0.42	0.55	35	[S14]
	Graphene/BN	Epoxy	0.20	11.01	6.02	1.83	5405	[S15]
	CF/VACNTs	PDMS	0.26	8.14	3.36	2.42	3031	[S16]
				7.51	3.72	2.02	2788	
				4.49	2.69	1.67	1627	

	RGO@MF	PDMS	0.26	0.76	1.68	0.45	192	[S17]
Random network	SWCNT	PVDF	0.23	0.28	0.28	1.00	22	[S18]
				0.31	0.31	1.00	35	
				0.37	0.37	1.00	61	
				0.45	0.45	1.00	96	
				0.48	0.48	1.00	109	
				0.54	0.54	1.00	135	
	CF/GNP	PCBT	0.19	4.00	5.60	0.71	2005	[S19]
				3.05	4.60	0.66	1505	
				1.65	2.35	0.70	768	
	GO/CNT	PVDF	0.23	1.13	1.13	1.00	391	[S20]
				1.56	1.56	1.00	578	
Orthotropic network	VSCG	PDMS	0.26	24.37	79.02	0.31	9273	This Work
				10.05	99.03	0.10	3765	
				6.34	113.61	0.06	2338	

Table S3 Elastic modulus and Poisson's ratio of each component of VSCG/PDMS composites

Sample		Elastic modulus (MPa)	Poisson's Ratio
VSCG-0.01/PDMS	CNT	2	0.03
	SiC	400	0.2
	HOGF	200	0.15
	PDMS	0.75	0.49
VSCG-0.03/PDMS	CNT	5	0.1
	SiC	400	0.2
	HOGF	200	0.15
	PDMS	0.75	0.49
VSCG-0.1/PDMS	CNT	30	0.15
	SiC	400	0.2
	HOGF	200	0.15
	PDMS	0.75	0.49

Table S4 The parameters of the cooling system used in Comsol simulation

	Size (mm^3)	ρ ($g\ cm^{-3}$)	C_p ($J\ g^{-1}\ K^{-1}$)	TC ($W\ m^{-1}\ K^{-1}$) //	⊥
Heater	15×15×1	3.90	0.91	27.00	27.00
Heat sink	120×55×7	8.96	0.39	400.00	400.00
VSCG-0.01/PDMS	15×15×0.3	0.89	1.32	79.02	24.37
T-FLEX 700	15×15×0.3	3.30	1.73	5.00	5.00

Table S5 The parameters and the calculated results of contact thermal resistance

Sample	BLT (μm)	k_{Bulk} ($W\ m^{-1}\ K^{-1}$)	k_{eff} ($W\ m^{-1}\ K^{-1}$)	R_c ($K\ mm^2\ W^{-1}$)
T-FLEX 700	300	5.00	1.90	97.89
VSCG-0.1/PDMS	300	24.37	7.75	26.40

Supplementary References

[S1] X. Shen, Z. Wang, Y. Wu, X. Liu, Y.-B. He et al., A three-dimensional multilayer graphene web for polymer nanocomposites with exceptional transport properties and fracture resistance. Mater. Horiz. **5**, 275–284 (2018). https://doi.org/10.1039/C7MH00984D

[S2] J. Chen, L. Wang, X. Gui, Z. Lin, X. Ke et al., Strong anisotropy in thermoelectric properties of CNT/PANI composites. Carbon **114**, 1–7 (2017). https://doi.org/10.1016/j.carbon.2016.11.074

[S3] A.M. Marconnet, N. Yamamoto, M.A. Panzer, B.L. Wardle, K.E. Goodson Thermal conduction in aligned carbon nanotube-polymer nanocomposites with high packing density. ACS Nano **5**, 4818–4825 (2011). https://doi.org/10.1021/nn200847u

[S4] Yan Q., F.E. Alam, Gao J., Dai W., Tan X. et al., Soft and self-adhesive thermal interface materials based on vertically aligned, covalently bonded graphene nanowalls for efficient microelectronic cooling. Adv. Funct. Mater. **31**, 2104062 (2021). https://doi.org/10.1002/adfm.202104062

[S5] C.-P. Feng, L. Bai, Y. Shao, R.-Y. Bao, Z.-Y. Liu et al., A facile route to fabricate highly anisotropic thermally conductive elastomeric POE/NG composites for thermal management. Adv. Mater. Interfaces **5**, 1700946 (2018). https://doi.org/10.1002/admi.201700946

[S6] H. Jung, S. Yu, N.S. Bae, S.M. Cho, R.H. Kim et al., High through-plane thermal conduction of graphene nanoflake filled polymer composites melt-processed in an L-shape kinked tube. ACS Appl. Mater. Interfaces **7**, 15256–15262 (2015). https://doi.org/10.1021/acsami.5b02681

[S7] P. Liu, X. Li, P. Min, X. Chang, C. Shu et al., 3D lamellar-structured graphene aerogels for thermal interface composites with high through-plane thermal conductivity and fracture toughness. Nano-Micro Lett. **13**, 22 (2020). https://doi.org/10.1007/s40820-020-00548-5

[S8] Min P., Liu J., Li X., An F., Liu P. et al., Thermally conductive phase change composites featuring anisotropic graphene aerogels for real-time and fast-charging solar-thermal energy conversion. Adv. Funct. Mater. **28**, 1805365 (2018). https://doi.org/10.1002/adfm.201805365

[S9] T. Tian, K.D. Cole, Anisotropic thermal conductivity measurement of carbon-fiber/epoxy composite materials. Int. J. Heat Mass Transf. **55**, 6530–6537 (2012). https://doi.org/10.1016/j.ijheatmasstransfer.2012.06.059

[S10] L. Jin, P. Wang, W. Cao, N. Song, P. Ding, Isolated solid wall-assisted thermal conductive performance of three-dimensional anisotropic MXene/graphene polymeric composites. ACS Appl. Mater. Interfaces **14**, 1747–1756 (2022). https://doi.org/10.1021/acsami.1c20267

[S11] J. Wang, T. Yang, Z. Wang, X. Sun, M. An et al., A thermochromic, viscoelastic nacre-like nanocomposite for the smart thermal management of planar electronics. Nanomicro Lett. **15**, 170 (2023). https://doi.org/10.1007/s40820-023-01149-8

[S12] J. Gong, Z. Liu, J. Yu, D. Dai, W. Dai et al., Graphene woven fabric-reinforced polyimide films with enhanced and anisotropic thermal conductivity. Compos. Part A Appl. Sci. Manuf. **87**, 290–296 (2016). https://doi.org/10.1016/j.compositesa.2016.05.010

[S13] Z. Jiang, T. Ouyang, L. Ding, W. Li, W. Li et al., 3D self-bonded porous graphite fiber monolith for phase change material composite with high thermal conductivity. Chem. Eng. J. **438**, 135496 (2022). https://doi.org/10.1016/j.cej.2022.135496

[S14] B. Li, S. Dong, X. Wu, C. Wang, X. Wang et al., Anisotropic thermal property of magnetically oriented carbon nanotube/graphene polymer composites. Compos. Sci. Technol. **147**, 52–61 (2017). https://doi.org/10.1016/j.compscitech.2017.05.006

[S15] F. An, X. Li, P. Min, H. Li, Z. Dai et al., Highly anisotropic graphene/boron nitride hybrid aerogels with long-range ordered architecture and moderate density for highly thermally conductive composites. Carbon **126**, 119–127 (2018).

https://doi.org/10.1016/j.carbon.2017.10.011

[S16] T. Ji, Y. Feng, M. Qin, S. Li, F. Zhang et al., Thermal conductive and flexible Silastic composite based on a hierarchical framework of aligned carbon fibers-carbon nanotubes. Carbon **131**, 149–159 (2018). https://doi.org/10.1016/j.carbon.2018.02.002

[S17] M. Qin, Y. Xu, R. Cao, W. Feng, L. Chen, Efficiently controlling the 3D thermal conductivity of a polymer nanocomposite via a hyperelastic double-continuous network of graphene and sponge. Adv. Funct. Mater. **28**, 1805053 (2018). https://doi.org/10.1002/adfm.201805053

[S18] Xu Y., G. Ray, B. Abdel-Magid, Thermal behavior of single-walled carbon nanotube polymer–matrix composites. Compos. Part A Appl. Sci. Manuf. **37**, 114–121 (2006). https://doi.org/10.1016/j.compositesa.2005.04.009

[S19] Y.J. Noh, S.Y. Kim, Synergistic improvement of thermal conductivity in polymer composites filled with pitch based carbon fiber and graphene nanoplatelets. Polym. Test. **45**, 132–138 (2015). https://doi.org/10.1016/j.polymertesting.2015.06.003

[S20] M. Cao, C. Du, H. Guo, X. Li, S. Song et al., Paving thermally conductive highway by 3D interconnected framework of carbon nanotube and graphene oxide in poly(vinylidene fluoride). Compos. Part A Appl. Sci. Manuf. **115**, 331–340 (2018). https://doi.org/10.1016/j.compositesa.2018.09.024
